# Aging and periodontitis increase brain dissemination of oral bacteria

**DOI:** 10.1002/jper.70118

**Published:** 2026-04-13

**Authors:** Ozge Unlu, Tsute Chen, Nil Yakar, Zeliha Guney, Bruce Paster, Isabel Carreras, Alpaslan Dedeoglu, Alpdogan Kantarci

**Affiliations:** ^1^ The ADA Forsyth Institute Cambridge Massachusetts USA; ^2^ Department of Medical Microbiology Istanbul Atlas University Faculty of Medicine Istanbul Turkey; ^3^ Department of Microbiology Ege University Izmir Turkey; ^4^ Department of Periodontology Ankara Medipol University Faculty of Dentistry Ankara Turkey; ^5^ Department of Oral Medicine Infection and Immunology Harvard University School of Dental Medicine Boston Massachusetts USA; ^6^ Department of Veterans Affairs VA Boston Healthcare System Boston Massachusetts USA; ^7^ Department of Neurology Boston University School of Medicine Boston Massachusetts USA; ^8^ Department of Biochemistry Boston University School of Medicine Boston Massachusetts USA; ^9^ Department of Radiology Massachusetts General Hospital and Harvard Medical School Boston Massachusetts USA; ^10^ Department of Developmental and Surgical Sciences University of Minnesota School of Dentistry Minneapolis Minnesota USA

**Keywords:** brain microbiome, inflammaging, oral microbiome, periodontitis, senescence

## Abstract

**Background:**

The microbiome is a dynamic system that changes throughout life. Studies have revealed the relationship between periodontal disease and the oral microbiota; however, the impact of periodontal disease on the expression of senescence markers and on the inflammaging of the oral and systemic microbiome remains unclear. We hypothesized that aging increases the periodontitis‐induced changes in the oral and systemic microbiome and is accompanied by an altered inflammatory response.

**Methods:**

Experimental periodontitis was induced in 18‐month‐old (old) and 8‐month‐old (young) C57BL/6 mice by placing ligatures around the second maxillary molars. Bone morphometric analyses were conducted to assess bone loss. Senescence‐ and inflammatory‐related gene expression in the gingiva was measured by quantitative polymerase chain reaction (qPCR). Serum inflammatory markers were evaluated via immunoassay. Oral, brain, and gut microbial content were analyzed using next‐generation sequencing.

**Results:**

Maxillary bone loss was significantly higher in the old mice with periodontal disease than in young mice. Senescence and inflammatory markers were higher in old mice than in young ones, and periodontitis increased their expression. The alpha diversity of the oral and brain microbial communities differed significantly between old and young mice. *Treponema denticola*, *Fusobacterium nucleatum, Porphyromonas gingivalis, P. pasteri*, and *Prevotella nigrescens* were only detected in the brains of old animals with periodontitis.

**Conclusion:**

Periodontopathogens and oral commensals are either only found in the brains of old animals with periodontal disease or are more prevalent in the brains of old animals, suggesting that aging and periodontitis may contribute to the dissemination of oral bacteria to the brain.

**Plain language summary:**

Aging may increase the periodontitis‐induced changes in the oral and systemic microbiome, which an altered inflammatory response may accompany. Experimental periodontitis was created in old and young mouse models. Bone loss, senescence, and inflammatory gene expression and serum inflammatory markers were assessed in each model, and oral, brain, and gut microbial content was analyzed. Senescence and inflammatory markers were higher in old mice than in young ones, and periodontitis increased their expression. Our results suggested that aging and periodontitis may contribute to the dissemination of oral bacteria to the brain.

## 1 INTRODUCTION

Inflammatory changes due to aging and dysregulation of the immune system lead to the production of pro‐inflammatory cytokines and acute‐phase proteins in older adults, even in the absence of infection. Inflammaging leads to a cycle that results in exacerbated inflammation and tissue damage, which consequently predisposes to age‐related diseases such as rheumatoid arthritis, atherosclerosis, periodontal diseases, and dementia.^1^, ^2^, ^3^ Aging is a risk factor for periodontal disease, involving aging‐related immune system cell dysfunctions in older adults, alterations in the oral and systemic microbiome, and inflammatory changes.^4^, ^5^ As in other systemic diseases and conditions, periodontitis is a source of infection and inflammatory changes. The impact of periodontal disease on inflammaging is unclear, yet the microbiome and the inflammatory changes could play a role in this bidirectional relationship.^6^, ^7^, ^8^

The oral cavity has the second most complex microbial composition in the human body.^9^, ^10^ Studies investigating the relationship between aging and the oral microbiome provided limited information at the phylum and genus levels; no studies have provided specific information at the species level.^10^, ^11^ Meanwhile, recent work demonstrated a strong bidirectional link between periodontal disease and neurodegenerative pathologies, including Alzheimer's disease and Parkinson's disease,^12^, ^13^, ^14^ potentially suggesting that periodontitis could be a local source of systemic dissemination of oral bacteria to the brain.^4^, ^5^ Indeed, oral bacteria, including *Fusobacteria* and spirochetes, and bacterial products, such as outer membrane vesicles and proteases from oral microorganisms (e.g., *Porphyromonas gingivalis*), have been recovered from the brain and linked to neurodegenerative processes.^15^, ^16^, ^17^ We showed that specific oral bacteria can reach the brain and activate microglial cells, supporting the role of oral bacteria in neuroinflammatory reactions and neurodegeneration. It is, however, unclear whether aging is the driving or a contributory factor in this association.^12^, ^13^, ^14^ Therefore, we hypothesized that aging increases the periodontitis‐induced changes in the oral microbiome, leading to the dissemination of periodontal bacteria to the brain, and that an altered inflammatory response accompanies this. We evaluated the impact of experimental periodontitis on the oral cavity and brain microbial profiles using next‐generation sequencing in young and old mice. To understand the relationship between inflammaging and periodontal disease, we investigated the level of senescence and inflammatory markers expressed in the gingiva.

## 2 MATERIALS AND METHODS

### 2.1 Animal model of experimental periodontitis

Eighteen‐month‐old (*n* = 32; 16 M, 16F, corresponding to older adults in humans) “old” mice and 8‐month‐old (*n* = 8; 4 M, 4F, corresponding to younger adults in humans) “young” C57BL/6 mice were purchased from Charles River Laboratories. Sample size was calculated through power analysis. The effect size was 0.8, and the alpha was at 0.05. In the old‐mice group, at least eight mice per sex per group were needed. All experimental mice and protocols were approved by the Institutional Animal Care and Use Committee (IACUC; approval number #22‐003) of The American Dental Association (ADA) Forsyth Institute. The study adhered to the ARRIVE reporting guidelines. Experimental periodontitis was induced by placing ligatures around the second maxillary molars for 30 days (*n* = 16; 8 M, 8F in old mice; *n* = 4; 2 M, 2F in young mice). Ligatures were renewed on the 15th day. All animals were sacrificed 30 days after the ligature placement.

### 2.2 Bone loss assessment

Periodontitis‐induced bone loss was quantified through morphometric analysis following the euthanasia and removal of the ligatures. The maxillae were cleaned of flesh by dermestid beetles over 4–5 days. The samples were then submerged in 5% hydrogen peroxide for 8 hours to ensure thorough cleaning and rinsed with water. To improve visualization, the samples were briefly stained with methylene blue (1% in water) for 10 seconds, which helped distinguish bone from dental structures before morphometric analysis. The specimens were then mounted and imaged at 10× magnification using an inverted microscope* and AxioVision 4.8 software. The area between the alveolar bone crest and the cementoenamel junction for each of the three maxillary molars was measured using ImageJ. The results were quantified in square millimeters.^18^, ^19^, ^20^, ^21^

### 2.3 Senescence and inflammatory profiles in gingiva

Gingival tissue surrounding the upper molars was gently removed using a scalpel blade under a microscope and stored in PRAS in a –80°C freezer.^22^ Gingiva samples were thawed and homogenized using a glass grinder. RNA extraction from the gingival samples was performed using the MasterPure Complete DNA and RNA Purification Kit. The concentration and the quality of RNA samples were assessed with a Nanodrop spectrophotometer. cDNA synthesis was performed with Invitrogen SuperScript IV VILO Master Mix†. Reverse transcription was carried out on a StepOne Real‐Time PCR (polymerase chain reaction) thermal cycler. The expression and relative quantification of p16 (CDKN2A), p53 (TP53), sirtuin 1‐5 (SIRT1‐5) senescence proteins, and interleukin (IL) ‐1β, tumor necrosis factor‐alpha (TNF‐α), and IL‐6 inflammatory markers were measured with quantitative PCR (qPCR).

### 2.4 Serum markers of inflammation

Blood was collected at the time of sacrifice, and serum was separated from the cellular fraction by centrifugation at 2000 *g* for 10 minutes. As a measure of the systemic inflammatory burden of the periodontitis model, serum levels of IL‐6, TNF‐α, and IL‐8 were measured using a multiplex immunoassay‡ according to the manufacturer's protocols. Data were evaluated against standard curves generated for each analyte and reported as picograms per milliliter.

### 2.5 DNA isolation and next‐generation sequencing of oral, brain, and gut microbiota

To determine the microbial content, DNA from the ligature, brain, and stool samples was isolated using the MasterPure DNA Purification Kit according to the manufacturer's instructions. DNA was suspended in 25 µL TE Buffer. DNA purity and concentration were measured and recorded using a NanoDrop spectrophotometer.

The DNA samples were prepared for targeted sequencing with the Quick‐16S Next Generation Sequencing (NGS) Library Prep Kit§. These primers were custom‐designed by Zymo Research to provide the best coverage of the 16S gene while maintaining high sensitivity. 16S V1‐V3 Primer Sequences (adapters not included): 27f (AGRGTTYGATYMTGGCTCAG, 20 bp) and 515r (TBACCGCGGCTGCTGGCAC, 19 bp). The sequencing library was prepared using an innovative library preparation process in which PCR reactions were performed in real‐time thermal cyclers to control cycles and limit PCR chimera formation. The final PCR products were quantified by fluorescence and pooled at equal molarity. The final pooled library was cleaned up with the Select‐a‐Size DNA Clean & Concentrator, then quantified with TapeStation and Qubit. The final library was sequenced on Illumina NextSeq 2000™ with a p1 reagent kit ‖ (600 cycles). The sequencing was performed with a 10% PhiX spike‐in. Absolute abundances were recorded, and bioinformatics analyses were performed.

### 2.6 Bioinformatics analysis of sequence data

Raw 16S V1V3 amplicon sequences obtained from Zymo Research were quality‐filtered, denoised, pair‐end merged, and chimera removed with the DADA2 tool (version 1.12.1).^23^ Amplicon sequence variants (ASVs) generated by DADA2 were subjected to species‐level taxonomy assignment based on the approach developed by Al‐Hebshi *et al*. ^24^ against the FOMC Reference Sequence Set (https://microbiome.forsyth.org/ftp/refseq) that consists of 1,015 full‐length 16S rRNA sequences from HOMD V15.22, 356 from MOMD V5.1, and 22,126 from NCBI, a total of 23,497 sequences. Altogether, these sequences represent 17,035 oral and non‐oral microbial species. Specie‐level read count tables were imported using R's “phyloseq” package (version 1.28.0) ^25^ for downstream analyses. Alpha diversity was calculated in three measurements: (1) number of species (observed), (2) Shannon index, and (3) Simpson index using the “phyloseq” package's “plot_richness” function.^25^

### 2.7 Read count statistics and rarefaction curves

After DADA2 quality filtering, denoising, pair merging, and chimera removal, the read count per sample ranges from 19,486 to 199,223 reads. These are enough sequencing depths to detect all species in the samples, as indicated by the rarefaction curves shown in Figure S1 and Table S1 in the online *Journal of Periodontology*.

### 2.8 Statistical analysis

qPCR data were analyzed using the Δ‐Δ‐CT method, and protein expression and bone loss were analyzed statistically using GraphPad Prism 4. Clinical morphometric data were analyzed using one‐way analysis of variance (ANOVA) with Sidak's multiple comparisons test. Alpha diversity was evaluated with QIIME2 (version 2020.11) “alpha‐group‐significance” function.^26^ To test whether the alpha diversity among different comparison groups differs statistically, we use the Kruskal–Wallis H test implemented by the “alpha‐group‐significance” function in the QIIME 2 “diversity” package. Beta diversity non‐metric multidimensional scaling (NMDS) plots were generated with the “ordinate” function in “phyloseq,” and beta diversity significance tests were evaluated with QIIME2 “beta‐group‐significance” function.^26^ ANCOMB‐BC2 (Analysis of Compositions of Microbiomes with Bias Correction) (version 2.0.2) was used to test the significance of differential abundance between two test groups.^27^ Linear discriminant analysis (LDA) effect size (LEfSe) (version 1.0.0) was used to plot the effect size of differentially abundant features.^28^

## 3 RESULTS

### 3.1 Aging affects periodontitis‐induced alveolar bone loss, senescence, and the inflammatory profile in gingival tissues

**FIGURE 1:**
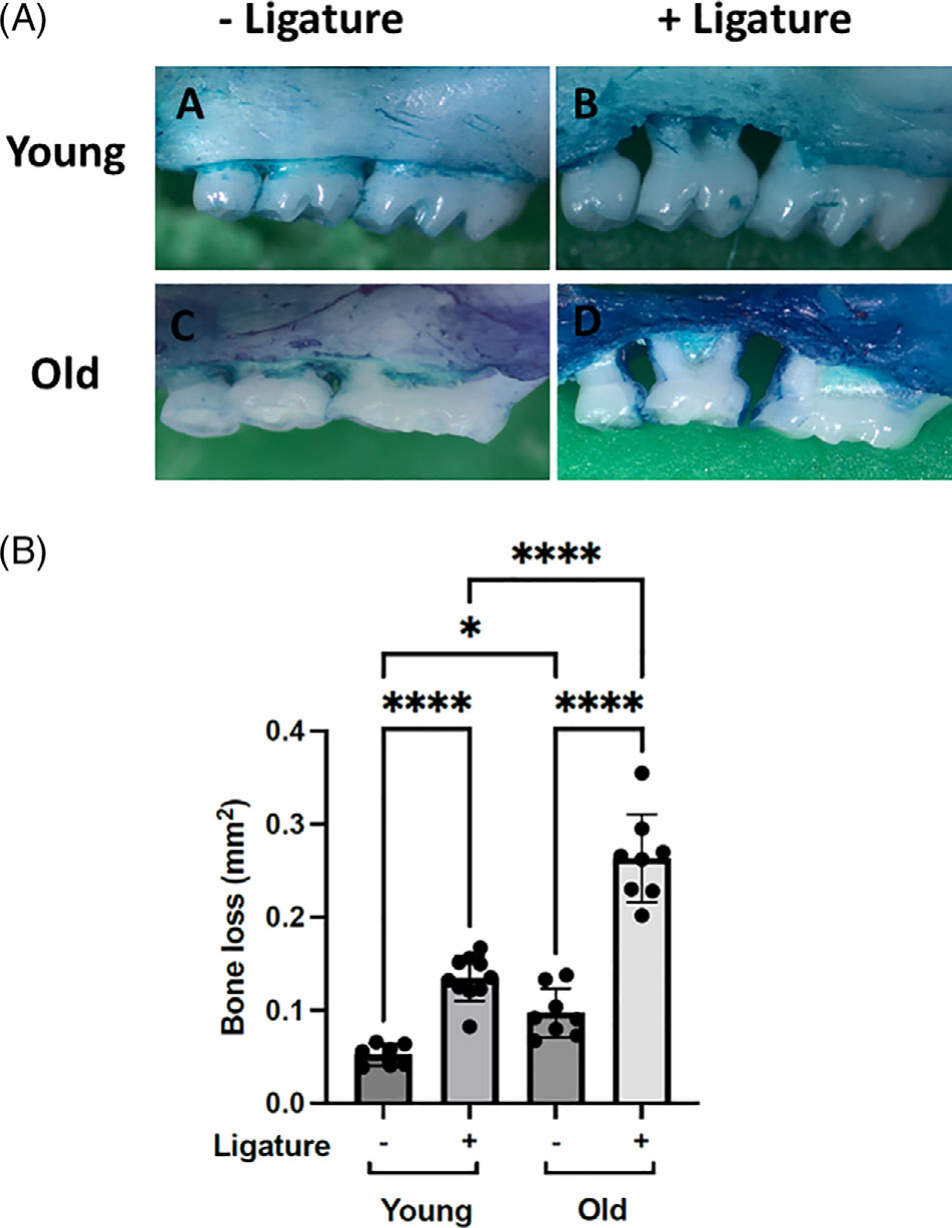
Top panel A: Representative images comparing the maxillary alveolar unit morphology in young non‐periodontitis (A), young periodontitis (B), old non‐periodontitis (C), and old periodontitis (D) mice. Bottom panel B: Mean bone loss of three molars, measured as the tooth surface area between the alveolar bone crest and the cementoenamel junction. NP, non‐periodontitis. *p < 0.05, ****p < 0.0001 (one‐way analysis of variance with Sidak's multiple comparisons test).

Maxillary bone loss was significantly greater in old mice with periodontal disease than in young mice (Figure 1). There were no significant differences according to sex. Senescence markers (p16 and p53) were expressed 2.7‐ and 1.2‐fold higher in old mice than in the young ones. Periodontitis led to a further increase in p16 and p53 in old mice. (Table 1). Sirtuin 1 and 5 were significantly decreased in old mice (25 and 3.2‐fold, respectively). Periodontitis led to a further decrease in sirtuin expression in old mice, collectively suggesting that senescence was associated with reduced anti‐aging markers in gingival tissues and that periodontitis exacerbated this process. In parallel, old mice had higher levels of TNF‐α and IL‐1β in their gingival tissues (13‐ and 11‐fold higher, respectively), while IL‐6 expression was reduced by four‐fold. Periodontitis further increased TNF‐α and IL‐1β (4.5‐ and 3.9‐fold, respectively) in old mice. IL‐6 was further decreased (1.6‐fold), suggesting that a disrupted inflammatory response accompanied the senescence‐associated changes in gingival tissues. No statistically significant changes were observed in serum pro‐inflammatory cytokines (TNF‐α, IL‐8, and IL‐6), suggesting that the systemic impact of periodontitis in young and old mice was not different (data not shown).

**TABLE 1:** Expression of senescence markers, sirtuins, and inflammatory markers in different groups.

	−PD			Old		
Parameters	Young	Old	Fold diff	−PD	+PD	Fold diff
p16	0.34	0.90	2.68	2.68	2.95	1.1
p53	1.41	1.66	1.17	1.66	1.73	1.05
Sirtuin 1	1.03	0.05	**0.04** *	0.05	0.03	0.83
Sirtuin 3	1.10	0.55	0.5	0.55	0.11	0.2
Sirtuin 5	1.05	0.33	**0.32** *	0.33	0.31	0.95
IL‐1β	1.06	11.69	**11** *	11.69	45.86	3.92
TNF‐α	1.02	13.35	13.10	13.35	59.67	4.47
IL‐6	1.00	0.25	0.25	0.25	0.15	0.6

Abbreviations: Fold diff, fold difference; −PD, without periodontitis; +PD, with periodontitis.

* *p* < 0.05.

### 3.2 Oral, gut, and brain microbial content in aging and the impact of periodontitis

**FIGURE 2:**
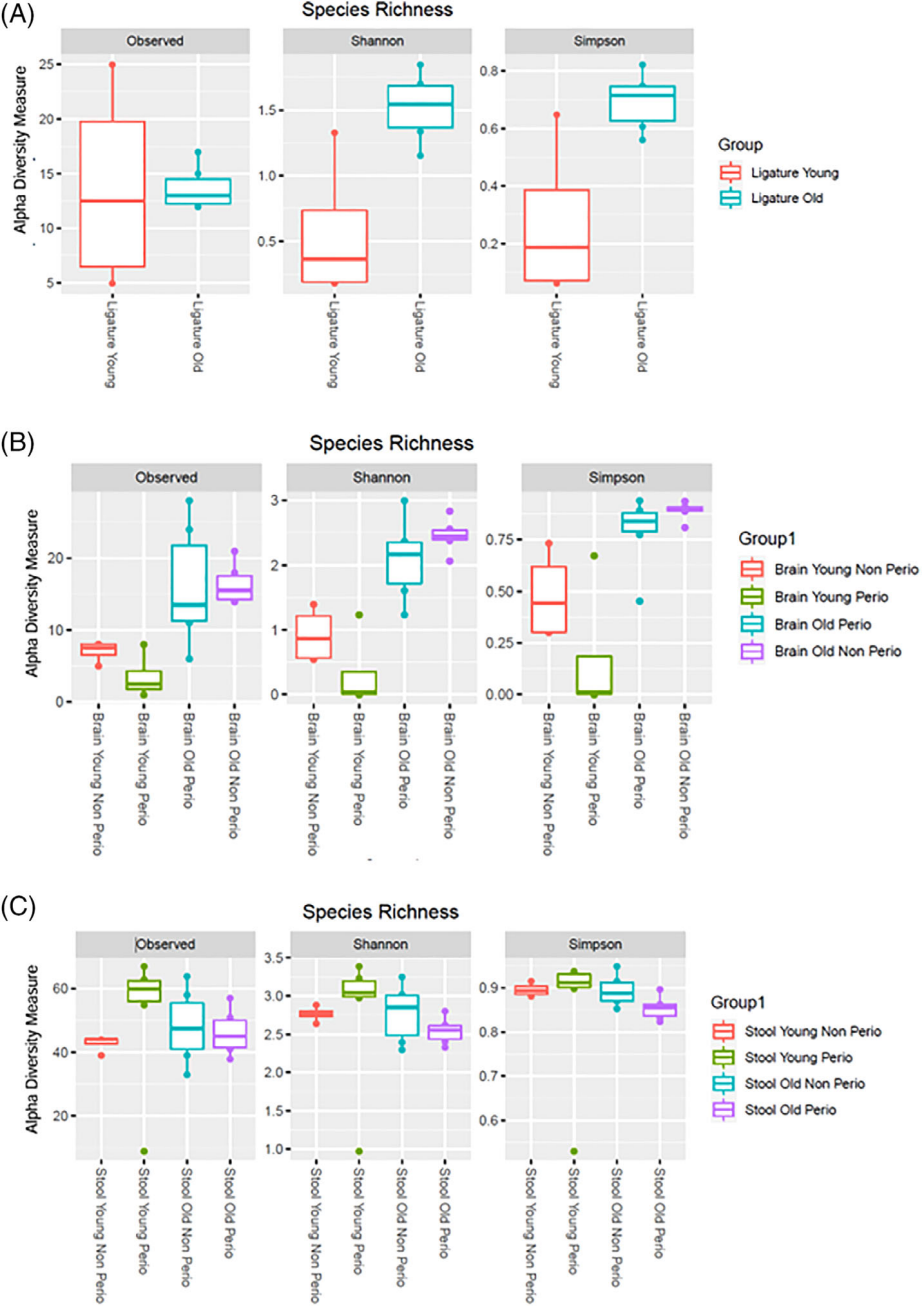
Alpha diversity according to Observed, Shannon, and Simpson Indexes. (A) Alpha diversity according to Observed, Shannon, and Simpson Indexes of ligature old vs young. (B) Alpha diversity according to Observed, Shannon, and Simpson Indexes of brain‐young‐nonperio vs. brain‐young‐perio vs. brain‐old‐nonperio vs. brain‐old‐perio. (C) Alpha diversity according to Observed, Shannon, and Simpson Indexes of stool‐young‐non perio vs stool‐young‐perio vs. stool‐old‐nonperio vs. stool‐old‐perio.

**FIGURE 3:**
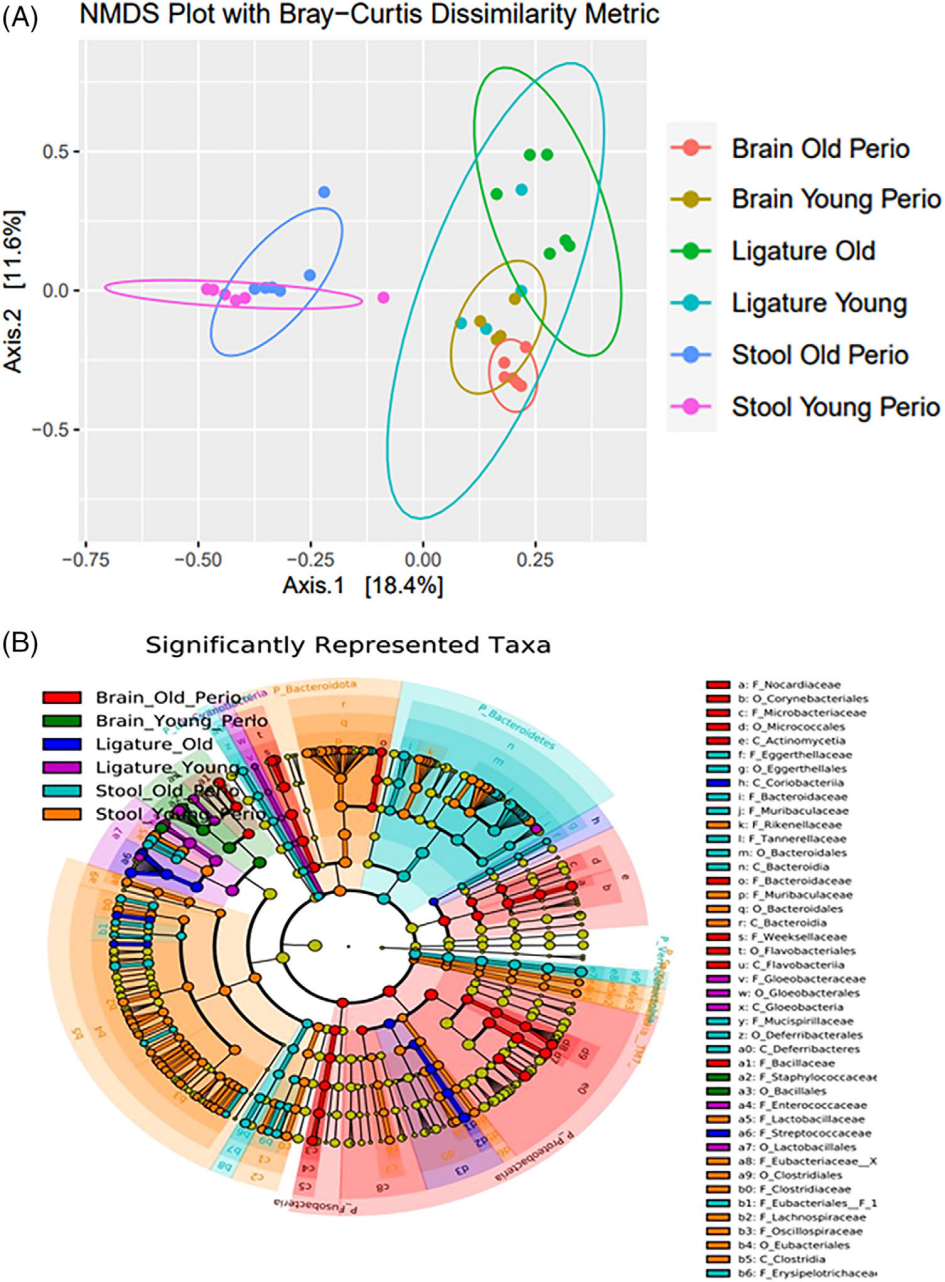
(A) Beta diversity NMDS plot with Bray‐Curtis dissimilarity metric. PERMANOVA results *p*‐value value = 0.001. (B) Significantly represented taxa. Number of significantly discriminative features: 244 (244) before internal Wilcoxon. Number of discriminative features with abs LDA score > 2.0: 244. NMDS, non‐metric multidimensional scaling; PERMANOVA, permutational multivariate analysis of variance.

NGS results revealed that the alpha diversity of the oral (Shannon index *p* = 0.019; Simpson index *p* = 0.033) and brain (Shannon index *p* = 0.0003, Simpson index *p* = 0.000517) microbial composition significantly differed between old and young mice (Figure 2), while the difference in gut microbiome alpha diversity was not statistically significant (*p* = 0.075). Periodontitis did not significantly affect alpha diversity in the brain in old (*p* = 0.11) or young (*p* = 0.15) animals, when comparing the periodontitis and non‐periodontitis subgroups. We also compared the alpha diversity among the brain, gut, and ligature microbiomes. Alpha diversity of the brain and gut microbiomes was significantly different in young animals with periodontitis (*p* = 0.02), suggesting distinct characteristics between these communities. The brain and ligature microbiomes were similar (*p* = 0.15), suggesting that the brain microbiome correlated with the oral microbiome in young animals with periodontitis. However, in old mice, the alpha diversity of the brain microbiome was not significantly different from either the stool (*p* = 0.08) or the oral (*p* = 0.11) microbiomes, suggesting that, with aging, brain microbial composition was influenced by both the gut and the oral microbiomes. Although the alpha diversity of the old mice's gut microbiome differed significantly by periodontal disease status (Simpson index, *p* = 0.03), a similar difference was not detected in the young mice (Simpson index, *p* = 0.40). These results were supported by the beta‐diversity analysis, which showed a permutational multivariate analysis of variance (PERMANOVA) *p*‐value of 0.01. The Bray‐Curtis dissimilarity metric showed distinct clustering of the brain, ligature, and gut microbiomes of old mice, whereas the ligature and brain microbiomes of young mice were not distinctly separated. These clusters are shown in Figure 3A, and the taxa that are significantly represented are shown in Figure 3B. Pairwise PERMANOVA results were provided as Table S2 in the online *Journal of Periodontology*.

### 3.3 Impact of sex on oral, gut, and brain microbial content

**FIGURE 4:**
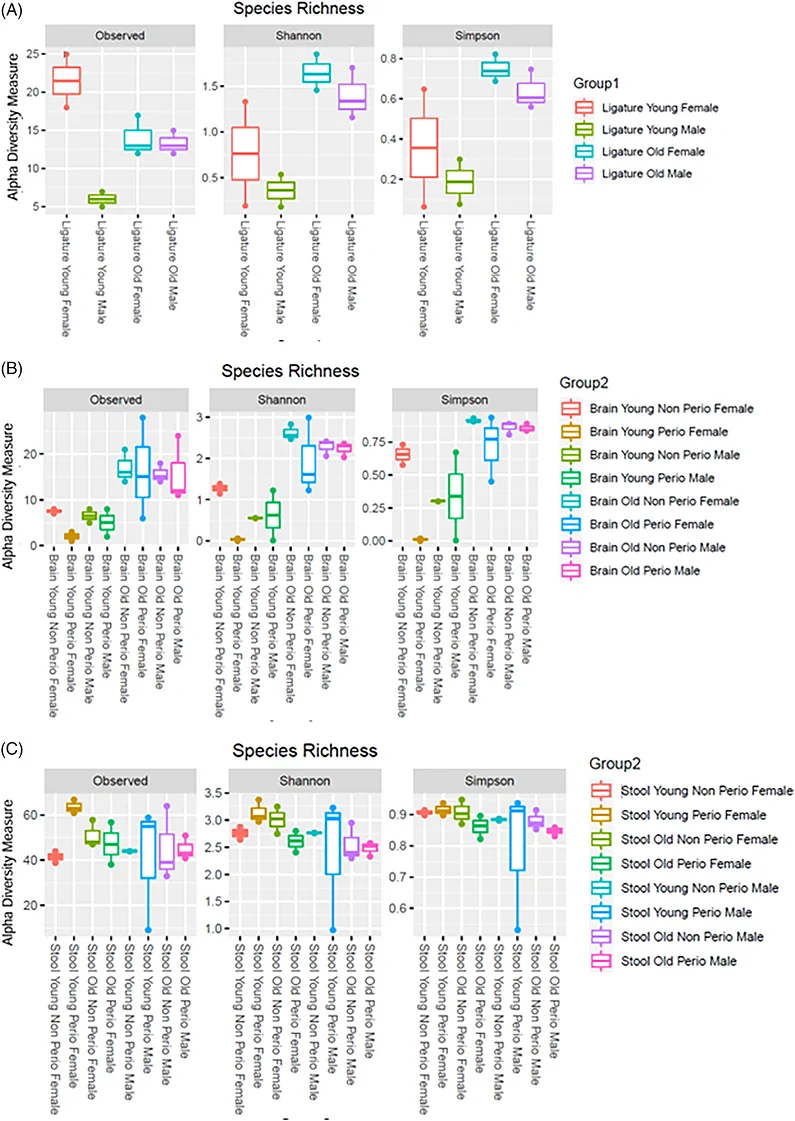
Alpha diversity according to Observed, Shannon, and Simpson Indexes in different sexes. (A) Ligature samples. (B) Brain samples. (C) Stool samples.

No significant differences were detected in the alpha diversity of the ligature microbiomes between sexes (Figure 4). In contrast, the alpha diversity of the brain microbiome in animals without periodontitis differed significantly between old female and old male mice (*p* = 0.04). However, no significant difference was observed between the brain microbiome of young female and male non‐periodontitis mice (*p* = 0.12). In the alpha diversity of the brain microbiome, no sex differences were observed in young (*p* = 0.44) or old (*p* = 0.51) animals with periodontitis, suggesting that periodontitis led to a relatively uniform brain microbiome by trafficking several species into the brain. Furthermore, no significant differences in gut microbiome alpha diversity were detected by sex across groups.

### 3.4 Significant taxa in oral, brain, and gut microbiomes impacted by periodontitis

At the species level, *Fusobacterium nucleatum*, considered a bridge species in the progression of the periodontal disease, was detected only in the brains of old animals and was three times more abundant in mice with periodontitis (Tables 2, 3, 4). *Treponema denticola* was detected only in the brains of old animals with periodontitis. *Porphyromonas pasteri, P. gingivalis*, and *Prevotella nigrescens*, among periodontopathogens, were occasionally detected in the brain samples of old animals with periodontitis. *Streptococcus mitis, S. salivarius*, and *Staphylococcus pasteri*, the members of the commensal oral microbiome, were detected only in the brains of old mice with periodontitis. *S. thoraltensis*, associated with endocarditis and bacteremia, was detected in samples from animals with periodontitis,^29^ in gut, ligature, and brain samples from old periodontitis mice, and in gut and brain samples from young periodontitis mice. These findings are presented in Table 2.

**TABLE 2:** Abundances of several oral bacteria in young and old mice with periodontitis.

Several bacterial species detected in the oral microbiome	Young +PD (%)	Old +PD (%)	Young vs old +PD fold diff
*Enterococcus faecalis*	37	1.2	30.83
*Escherichia coli*	2	0	
*Bifidobacterium pseudolongum*	2	0.01	200.00
*Citrobacter koseri*	5	6.9	0.72
*Enterobacter cloacae*	0	0.4	
*Enterobacter hormaechei*	20.6	23.2	0.89
*Kosakonia sacchari*	0	4.5	
*Streptococcus danieliae*	0.28	0.03	9.33
*Ligilactobacillus murinus*	9.3	1.6	5.81
*Streptococcus thoraltensis*	0	9	

Abbreviations: fold diff, fold difference; +PD, with periodontitis.

*Bifidobacterium pseudolongum*, known for its probiotic properties, was detected 6.7 and 6.3‐fold less in the stool of old and young animals with periodontitis, respectively, compared to their non‐periodontitis controls. This species was also occasionally found in ligature samples from old animals, at levels 200 times higher than in young animals. *Ligilactobacillus murinus*, another probiotic bacterium, was detected 3.5‐fold lower in stool samples of young animals with periodontitis than in those of non‐periodontitis. It was 5.8‐fold higher in ligature samples from young animals than in those from old animals. In contrast, it was 1.5‐fold more abundant in the stool samples of old animals with periodontitis than in those of non‐periodontitis animals. *Rhodococcus qingshengii* was detected only in the brains of old animals, two‐fold higher in the periodontitis group. *Haemophilus haemolyticus*, a commensal bacterium that colonizes the respiratory tract, was detected in the brains of old animals with periodontitis.

*Klebsiella pneumoniae* was detected in young animals with periodontitis, and all brain samples isolated from old animals, even in the absence of periodontitis. It was twice as common in the brains of animals with periodontitis. *Enterococcus faecalis* was detected in the brains of young animals with periodontitis and in old animals with or without periodontitis, with a 3.2‐fold increase in abundance in the periodontitis group. It was found to be 2.5 times higher in the brains of young animals with periodontitis than in those of older animals. In ligature samples, it was detected to be 30 times higher in young animals than in old animals. *Proteus mirabilis* was detected only in brain samples from old mice, with a five‐fold increase in the periodontitis group. Its abundance was 1.7 times higher in female brains. *Enterobacter hormaechei*, a member of the gastrointestinal tract, was detected 27.5 times more in the stool samples of old animals with periodontitis and three times more in their brains than in healthy animals. *E. hormaechei* was not detected in any stool samples of young animals, while it was detected in brain samples, with higher levels in those with periodontitis. It was detected in ligature samples from both young and old animals, with a 2.6% increase in the old ones. *Enterobacter cancerogenus*, another commensal member of the intestinal microbiome, was detected only in samples from old animals. Its abundance was 28‐fold higher in the brains of animals with periodontitis. *Shigella flexneri* and *S. sonnei* were detected only in old animal brain samples, and *S. sonnei* was detected four times more abundantly in animals with periodontitis. *Enterocloster bolteae* was detected only in fecal and ligature samples from old animals, and its abundance was eight times higher in stool samples from those with periodontitis. *Enterobacter cloacae* was detected only in old animals, and there was no difference in its abundance in brain samples between periodontitis and healthy animals.

*Citrobacter koseri* was detected 33‐fold higher in the stool of old mice with periodontitis than in healthy mice. *C. koseri* was detected in the brains of young mice, both healthy and those with periodontitis, being 2.4‐fold higher than in those without periodontitis. *Escherichia coli* was detected 1.7‐ and two‐fold higher in the stool and brain samples of young animals with periodontitis than in healthy animals. Interestingly, *E. coli* was not detected in any of the old mice. The gut microbiome in aging and the impact of periodontitis are shown in Table 3, and the brain species are shown in Table 4. Different patterns were observed in female and male mice for ligature, brain, and stool samples. While *Staphylococcus* was the most abundant genus in ligature samples from female mice, followed by Streptococcus, Enterobacter was the most prevalent genus in male samples, followed by *Alistipes*. *F. nucleatum* levels were 8.3‐fold, and *S. sonnei* levels were 6.6‐fold higher in the male brains than the female brains with periodontitis. *P. mirabilis* was 1.7 times higher in female brains, while *K. pneumoniae* levels were detected as 4.6‐fold higher in the brains of males.

**TABLE 3:** Abundances of several bacterial species in the gut microbiome of young and old mice with or without periodontitis.

	Young			Old			Young vs old	
Several bacterial species detected in the gut microbiome	−PD (%)	+PD (%)	Fold diff	−PD (%)	+PD (%)	Fold diff	−PD fold diff	+PD fold diff
*Enterococcus faecalis*	0	0		0.5	0.3	−1.7		
*Escherichia coli*	9.5	15.7	1.65	0	0			
*Bifidobacterium pseudolongum*	0.19	0.03	−6.3	0.2	0.03	−6.7	1.1	1
*Citrobacter koseri*	0	0		0.06	2	33.3		
*Cutibacterium acnes*	0	16.3		0	0			
*Enterobacter cancerogenus*	0	0		0	0.1			
*Enterobacter cloacae*	0	0		0	0.03			
*Enterobacter hormaechei*	0	0		0.24	6.6	27.5		
*Lactobacillus johnsonii*	0	0		0.06	0.44	7.3		
*Shigella flexneri*	4.2	7.6	1.8	0	0			
*Shigella sonnei*	4.2	7.6	1.8	0	0			
*Streptococcus thoraltensis*	0	11.87		0	0.004			−2950
*Enterocloster bolteae*	0	0		0.006	0.05	8.3		
*Enterococcus gallinarum*	0	0		0	0.3			
*Faecalibaculum rodentium*	0	0		0.06	0.35	5.8		
*Lactobacillus intestinalis*	0	0		0.54	0.44	−1.22		
*Ligilactobacillus murinus*	3.2	0.9	−3.5	7.7	11.3	1.5	2.4	12.5

Abbreviations: fold diff, fold difference; −PD, without periodontitis; +PD, with periodontitis.

**TABLE 4:** Comparison of the brain dissemination of microorganisms in young and old mice with or without periodontitis.

	Young			Old			Young vs old	
Several bacterial species detected in the brain	−PD (%)	+PD (%)	Fold diff	−PD (%)	+PD (%)	Fold diff	−PD fold diff	+PD fold diff
*Acinetobacter johnsonii*	4.2	6.8	1.62	3.1	4.2	1.35	−1.35	−1.62
*Enterococcus faecalis*	0	0.6		0.08	0.26	3.25		−2.31
*Escherichia coli*	14	30.3	2.16	0	0			
*Bifidobacterium pseudolongum*	0	0		0	0.76			
*Citrobacter koseri*	0.95	2.26	2.38	0	0.4			−5.65
*Cutibacterium acnes*	2	8	4	8.7	1.5	−5.8	4.35	−5.33
*Enterobacter cancerogenus*	0	0		0.01	0.28	28		
*Enterobacter cloacae*	0	0		0.7	0.75	1.1		
*Enterobacter hormaechei*	12.8	14.3	1.12	0.6	2	3.33	−21.33	−7.15
*Shigella flexneri*	0	0		1.8	0.6	−3		
*Shigella sonnei*	0	0		0.27	1.2	4.44		
*Streptococcus thoraltensis*	0	0		0	0.09			
*Acinetobacter lwoffii*	0.78	2.89	3.7	2.2	0.33	−6.7	2.82	−8.75
*Fusobacterium nucleatum*	0	0		1	3	3		
*F.nucleatum subsp.vincentii*	0	0		1.6	0.98	−1.6		
*Fusobacterium periodonticum*	0	0		1.1	0.2	−5.5		
*Gemella morbillorum*	0	0		0.6	0.1	−6		
*Haemophilus haemolyticus*	0	0		0	0.18			
*Klebsiella pneumoniae*	0	2.5		4.5	8.8	1.95		3.52
*Porphyromonas endodontalis*	0	0		2	0.2	−10		
*Porphyromonas gingivalis*	0	0		0	0.27			
*Porphyromonas pasteri*	0	0		0	0.27			
*Prevotella nigrescens*	0	0		0	0.22			
*Proteus mirabilis*	0	0		4	20.7	5.17		
*Rhodococcus qingshengii*	0	0		2.7	4.2	1.55		
*Streptococcus mitis*	0	0		0	0.14			
*Streptococcus salivarius*	0	0		0	0.11			
*Staphylococcus pasteri*	0	0		0	0.18			
*Streptococcus mutans*	0	0		1	0			
*Streptococcus oralis*	0	0		2.9	0			
*Treponema denticola*	0	0		0	1.2			

Abbreviations: fold diff, fold difference; −PD, without periodontitis; +PD, with periodontitis.

## 4 DISCUSSION

Inflammaging is chronic inflammation caused by aging and involved in age‐related diseases such as periodontitis.^1^, ^3^ The oral microbiome is a dynamic system that changes throughout life. There are very few studies investigating the impact of aging on the oral microbiome.^9^, ^10^, ^11^ Although studies have revealed the relationship between periodontal disease and the oral microbiota, the impact of aging on this association remains unclear.^4^, ^5^, ^12^, ^13^, ^14^, ^30^ Therefore, we hypothesized that aging would increase the periodontitis‐induced changes in the oral and systemic microbiomes, accompanied by an altered inflammatory response.

Our results demonstrated that periodontal disease increased bone loss in old mice regardless of sex. Senescence proteins p16, a cyclin‐dependent kinase inhibitor in the p16/Rb pathway that blocks G1‐S progression and leads to cell cycle arrest, and p53, a tumor suppressor that controls cellular senescence, were expressed at higher levels in old mice, confirming the impact of aging on periodontal tissues. Notably, periodontitis was associated with increased expression of senescence markers, suggesting that the inflammation associated with periodontal disease exacerbated aging‐associated changes. Similar to our results, Rattanaprukskul et al. reported increased gingival p16 gene expression in both young and aged subjects with periodontitis compared with healthy groups in their study using gingival biopsies.^31^ They validated enhanced senescence features during periodontitis by demonstrating significantly elevated gingival tissue SA‐β‐galactosidase activity in periodontal lesions of both age groups compared to age‐matched healthy sites.^31^ Thus, our model in mice replicated the human findings and suggested that periodontitis can accelerate aging in periodontal tissues in both mice and humans.

Sirtuins, which are associated with many metabolic activities such as DNA repair, vascular protection, cell‐cycle control, promoting longevity, protecting against cancer, apoptosis, and intracellular signaling, were detected at higher levels in young mice than in the old ones, where periodontitis decreased sirtuin expression, further demonstrating the impact of periodontitis on tissue senescence.^32^ In parallel, inflammatory markers such as IL‐1β and TNF‐α were elevated in gingival samples from old mice with periodontitis compared with those from healthy mice. IL‐1β and TNF‐α were detected at 11‐ and 13‐fold higher levels in the old compared to the young mice, supporting the “inflammaging” hypothesis and suggesting that older individuals may be more susceptible to periodontal disease. Interestingly, IL‐6 levels were lower in old mice than in young mice and among old mice with periodontitis. Collectively, these data confirmed the impact of aging and periodontitis on gingival tissues. Recent studies have demonstrated common molecular characteristics of periodontitis pathophysiology, including senescence‐associated secretory phenotype (SASP) and inflammation.^31^, ^33^, ^34^, ^35^ Guo et al. demonstrated the elevated level of cellular senescence in fibroblasts and endothelial cells from inflamed gingival tissue using single‐cell RNA sequencing. They showed an increased proportion of SA‐β‐gal‐positive cells, enlarged cell morphology, and significant upregulation of p16INK4A expression in inflamed human gingival tissues compared with healthy human gingival tissues.^36^

After characterizing the senescence and inflammaging, we focused on elucidating the oral and brain microbial content in aging and periodontitis. NGS data revealed that aging and periodontal disease significantly altered the microbial communities of oral, gastrointestinal, and brain tissues. The data suggested that aging and periodontal disease facilitated the migration of oral bacteria, including pathogens and commensals, into the brain. The levels of *B. pseudolongum*, classified as a probiotic, declined with age and decreased with periodontal disease in the gut and oral microbiomes. While the abundance of *L. murinus*, another probiotic bacterium, declined in the gut microbiome with periodontal disease among young animals, its levels were 5.8‐fold less in the ligature samples obtained from old mice than the young ones, suggesting the adverse impact of aging and periodontitis on the beneficial bacteria in both oral and gut microbiomes.

*K. pneumoniae, P. mirabilis*, and *E. faecalis* were only found in the brains of young animals that had periodontitis. In contrast, they were found in older animals at higher abundance than in those with periodontitis. Likewise, *E. cancerogenus* and *S. sonnei*, intestinal bacteria, were detected only in the brains of old mice and were 35‐fold and five‐fold higher in those with periodontitis, respectively. Sex‐based microbiome patterns were observed. *F. nucleatum, S. sonnei*, and *K. pneumoniae* levels were detected as 8.3, 6.6, and 4.6‐fold higher in the brains of males than in the female brains with periodontitis, while *P. mirabilis* was 1.7 times higher in female brains than in male brains. Periodontopathogens may directly trigger aging.^33^, ^34^, ^37^, ^38^ Long‐term exposure to the periodontal pathogen *F. nucleatum*, which is linked to many comorbidities, has been shown to cause gingival keratinocytes to exhibit senescence‐like characteristics, such as increased p16 and p21 levels and SA‐β‐Gal activity, which, in turn, reduces their capacity for regeneration.^33^ Aquino‐Martinez et al. indicated that the expression of senescent markers such as p16, p21, and p53 increased, and senescent osteocytes clustered in the alveolar bone in response to the oxidative environment caused by a prolonged bacterial challenge, with a detrimental effect on periodontal homeostasis by exacerbating chronic inflammation and hindering regeneration in old mice.^37^ *Aggregatibacter actinomycetemcomitans* cytolethal distending toxin (CDT), a bacterial genotoxin that promotes tissue colonization in part by modifying the host immune system, can directly damage DNA and induce senescence‐associated characteristics in a variety of cell types.^39^ Thus, aging is a recognized risk factor for periodontitis, and periodontitis may also trigger senescence, suggesting a reciprocal relationship between aging and periodontal disease, where each process may impact the other bi‐directionally.

The term “microbiome” may not apply to the brain, as there is no evidence of an organized microbial structure. Therefore, we refrained from using the term ‘microbiome’ to refer to microbial species detected in the brain. The distinction between old vs. young or PD+ vs. PD‐ suggested that the mentioned organisms may not be present in the normal state, and that conditions such as aging and periodontal disease may affect the brain's microbial content. *P. gingivalis* has been linked to many diseases in humans, and several studies have revealed its potential to migrate to the brain. For example, Iada et al. detected *P. gingivalis* in the spinal fluid of a 57‐year‐old woman with a brain abscess.^40^ In addition, Yoo et al. reported a case of *P. gingivalis* causing brain abscess in a patient with recurrent periodontitis.^41^ Thus, the “common belief” that *P. gingivalis* is a strictly human species and not found in mice has been challenged and supported by the “Mouse Oral Microbiome Database”¶.^42^ Meanwhile, the outer membrane vesicles (OMVs) of *P. gingivalis* have been repeatedly linked to several non‐oral diseases, including neurodegenerative pathologies.^42^, ^43^ This observation does not necessarily require “live” species to be detected in the brain, and it challenges the assumption that this specific human periodontopathobiont does not reach the brain and get involved in non‐oral diseases in non‐human species. Indeed, we (and others) could not recover “live” *P. gingivalis* from the brain. This observation would also support the detection of *P. gingivalis* at the DNA level, but not of living organisms. This makes sense, given that an aerobic brain is unfavorable to anaerobic species, including *P. gingivalis*. Nevertheless, we were puzzled by the detection of *P. gingivalis* in the brain and not around the teeth, possibly due to the low DNA concentration in the ligature samples. Detecting *P. gingivalis* in the brains of the aged mice was a reproducible observation.

This study has a limited sample size because obtaining aged mice and generating sufficient microbial samples for sequencing are major challenges. Due to the fragility of 18‐month‐old mice and the difficulty in keeping them alive until the end of the experiments, ligatures were used for only 30 days in the periodontitis model. Nevertheless, significant differences between old and young mice and the impact of periodontitis could be measured, suggesting profound changes due to aging and periodontitis. Another important finding was the observation of increased trafficking of oral bacteria to the brain in old mice in experimental periodontitis. Therefore, although the sample size was limited, the effects were significant and correlated with measures of senescence.

## 5 CONCLUSION

Aging leads to irreversible maxillary bone loss, increased expression of senescence proteins, inflammation markers in gingival tissues, and decreased production of sirtuins. Periodontal disease exacerbates these conditions and contributes to the adverse effects of aging. Aging and periodontal diseases may alter the oral, gastrointestinal, and brain microbiomes. Periodontopathogens and oral commensals are detected only in the brains of old animals, and they are more abundant in those with periodontal disease, suggesting that aging and periodontitis may be contributing to the dissemination of oral bacteria to the brain.

## AUTHOR CONTRIBUTIONS

Ozge Unlu, Nil Yakar, and Zeliha Guney performed the in vivo and in vitro experiments and their laboratory analyses. Tsute Chen and Bruce Paster performed the bioinformatics analyses. Alpdogan Kantarci and Ozge Unlu analyzed the data and wrote the manuscript. Isabel Carreras, Alpaslan Dedeoglu, and Bruce Paster designed the study and revised the manuscript. All authors approved the final version of the manuscript.

## CONFLICT OF INTEREST STATEMENT

The authors have no conflicts of interest to declare.

## ETHICAL APPROVAL

The Institutional Animal Care and Use Committee (IACUC) of the ADA Forsyth Institute reviewed and approved the experimental protocols.

## Supporting information



Supporting Information

Supporting Information

Supporting Information

## Data Availability

The data that support the findings of this study are available on request from the corresponding author (AK).

## Footnotes

* Axiovert 200, Zeiss, Thornwood, NY, USA

† Thermo Fischer Scientific, Waltham, MA, USA

‡ Milliplex, Millipore Corporation, Bedford, MA, USA

§ Zymo Research, Irvine, CA, USA

‖ Illumina, San Diego, CA, USA

¶
https://momd.org

## References

[1] Sendama W . The effect of ageing on the resolution of inflammation. Ageing Res Rev. 2020;57:101000. 10.1016/j.arr.2019.101000 31862417 PMC6961112

[2] Pawelec G , Goldeck D , Derhovanessian E . Inflammation, ageing and chronic disease. Curr Opin Immunol. 2014;29:23‐28. 10.1016/j.coi.2014.03.007 24762450

[3] Franceschi C , Bonafè M , Valensin S , et al. Inflamm‐aging. An evolutionary perspective on immunosenescence. Ann N Y Acad Sci. 2000;908:244‐254. 10.1111/j.1749-6632.2000.tb06651.x 10911963

[4] Paksoy T , Ustaoğlu G , Yaman D , et al. The link between total antioxidant status, total oxidant status, arylesterase activity, and subgingival microbiota in psoriasis patients. Int J Dermatol. Published online July 30, 2022. 10.1111/ijd.16353 35906956

[5] Paksoy T , Ustaoglu G , Tasci M , Demirci M , Unlu O , Yasar MF . Assessment of Epstein‐Barr virus, Candida albicans, and some periodontal pathogens in rheumatoid arthritis patients with periodontitis. North Clin Istanb. 2023;10(4):490‐500. 10.14744/nci.2023.58998 37719252 PMC10500248

[6] Lamparello AJ , Namas RA , Abdul‐Malak O , Vodovotz Y , Billiar TR . Young and aged blunt trauma patients display major differences in circulating inflammatory mediator profiles after severe injury. J Am Coll Surg. 2019;228(2):148‐160. 10.1016/j.jamcollsurg.2018.10.019 30448299 PMC7768550

[7] Ferrucci L , Corsi A , Lauretani F , et al. The origins of age‐related pro‐inflammatory state. Blood. 2005;105(6):2294‐2299. 10.1182/blood-2004-07-2599 15572589 PMC9828256

[8] Ebersole JL , Graves CL , Gonzalez OA , et al. Aging, inflammation, immunity and periodontal disease. Periodontol 2000. 2016;72(1):54‐75. 10.1111/prd.12135 27501491

[9] Belibasakis GN . Microbiological changes of the ageing oral cavity. Arch Oral Biol. 2018;96:230‐232. 10.1016/j.archoralbio.2018.10.001 30308473

[10] Zhang Y , Wang X , Li H , Ni C , Du Z , Yan F . Human oral microbiota and its modulation for oral health. Biomed Pharmacother. 2018;99:883‐893. 10.1016/j.biopha.2018.01.146 29710488

[11] Martino C , Dilmore AH , Burcham ZM , Metcalf JL , Jeste D , Knight R . Microbiota succession throughout life from the cradle to the grave. Nat Rev Microbiol. Published online July 29,2022:1‐14. 10.1038/s41579-022-00768-z PMC12875531 35906422

[12] Yilmaz M , Yay E , Balci N , et al. Parkinson's disease is positively associated with periodontal inflammation. J Periodontol. 2023;94(12):1425‐1435. 10.1002/JPER.23-0274 37433175

[13] Yay E , Yilmaz M , Toygar H , et al. Oral and gut microbial profiling in periodontitis and Parkinson's disease. J Oral Microbiol. 2024;16(1):2331264. 10.1080/20002297.2024.2331264 38528960 PMC10962298

[14] Yay E , Yilmaz M , Toygar H , et al. Parkinson's disease alters the composition of subgingival microbiome. J Oral Microbiol. 2023;15(1):2250650. 10.1080/20002297.2023.2250650 37649970 PMC10464550

[15] Pisani F , Pisani V , Arcangeli F , Harding A , Singhrao SK . The mechanistic pathways of periodontal pathogens entering the brain: the potential role of *Treponema denticola* in tracing Alzheimer's disease pathology. Int J Environ Res Public Health. 2022;19(15):9386. 10.3390/ijerph19159386 35954742 PMC9368682

[16] Parra‐Torres V , Melgar‐Rodríguez S , Muñoz‐Manríquez C , et al. Periodontal bacteria in the brain—Implication for Alzheimer's disease: a systematic review. Oral Dis. 2023;29(1):21‐28. 10.1111/odi.14054 34698406

[17] Li R , Wang J , Xiong W , et al. The oral‐brain axis: can periodontal pathogens trigger the onset and progression of Alzheimer's disease?. Front Microbiol. 2024;15. 10.3389/fmicb.2024.1358179 PMC10868393 38362505

[18] Yakar N , Unlu O , Cen L , et al. Targeted elimination of Fusobacterium nucleatum alleviates periodontitis. J Oral Microbiol. 2024;16(1):2388900. 10.1080/20002297.2024.2388900 39139835 PMC11321114

[19] Almarhoumi R , Alvarez C , Harris T , et al. Microglial cell response to experimental periodontal disease. J Neuroinflammation. 2023;20(1):142. 10.1186/s12974-023-02821-x 37316834 PMC10265806

[20] Nashef A , Qabaja R , Hazan R , et al. The collaborative cross‐mouse population for studying genetic determinants underlying alveolar bone loss due to polymicrobial synergy and dysbiosis. Int J Mol Sci. 2024;25(1):473. 10.3390/ijms25010473 PMC10778843 38203644

[21] Oner F , Kantarci A . Periodontal response to nonsurgical accelerated orthodontic tooth movement. Periodontol 2000. Published online January 22, 2025. 10.1111/prd.12623 39840535

[22] Abdalla HB , Alvarez C , Wu YC , et al. Soluble epoxide hydrolase inhibition enhances production of specialized pro‐resolving lipid mediator and promotes macrophage plasticity. Br J Pharmacol. 2023;180(12):1597‐1615. 10.1111/bph.16009 36508312 PMC10175184

[23] Callahan BJ , McMurdie PJ , Rosen MJ , Han AW , Johnson AJA , Holmes SP . DADA2: high‐resolution sample inference from Illumina amplicon data. Nat Methods. 2016;13(7):581‐583. 10.1038/nmeth.3869 27214047 PMC4927377

[24] Al‐Hebshi NN , Nasher AT , Idris AM , Chen T . Robust species taxonomy assignment algorithm for 16S rRNA NGS reads: application to oral carcinoma samples. J Oral Microbiol. 2015;7. 10.3402/jom.v7.28934 PMC4590409 26426306

[25] McMurdie PJ , Holmes S . Phyloseq: an R package for reproducible interactive analysis and graphics of microbiome census data. PLoS ONE. 2013;8(4):e61217. 10.1371/journal.pone.0061217 23630581 PMC3632530

[26] Bolyen E , Rideout JR , Dillon MR , et al. Reproducible, interactive, scalable and extensible microbiome data science using QIIME 2. Nat Biotechnol. 2019;37(8):852‐857. 10.1038/s41587-019-0209-9 31341288 PMC7015180

[27] Lin H , Peddada SD . Analysis of compositions of microbiomes with bias correction. Nat Commun. 2020;11(1):3514. 10.1038/s41467-020-17041-7 32665548 PMC7360769

[28] Segata N , Izard J , Waldron L , et al. Metagenomic biomarker discovery and explanation. Genome Biol. 2011;12(6):R60. 10.1186/gb-2011-12-6-r60 21702898 PMC3218848

[29] Chiorescu RM , Buksa SB , Botan A , Mocan M , Costache C , Toc DA . Vancomycin‐resistant *Streptococcus thoraltensis*: a case report of bacterial endocarditis and review of literature on infections caused by this pathogen. Microorganisms. 2024;12(3):566. 10.3390/microorganisms12030566 38543617 PMC10975471

[30] Unlu O , Demirci M , Paksoy T , et al. Oral microbial dysbiosis in patients with oral cavity cancers. Clin Oral Investig. 2024;28(7):377. 10.1007/s00784-024-05770-8 PMC11182825 38884817

[31] Rattanaprukskul K , Xia XJ , Jiang M , Albuquerque‐Souza E , Bandyopadhyay D , Sahingur SE . Molecular signatures of senescence in periodontitis: clinical insights. J Dent Res. 2024;103(8):800‐808. 10.1177/00220345241255325 38877743 PMC11308264

[32] Zhao L , Cao J , Hu K , et al. Sirtuins and their biological relevance in aging and age‐related diseases. Aging Dis. 2020;11(4):927. 10.14336/AD.2019.0820 32765955 PMC7390530

[33] Albuquerque‐Souza E , Shelling B , Jiang M , Xia XJ , Rattanaprukskul K , Sahingur SE . Fusobacterium nucleatum triggers senescence phenotype in gingival epithelial cells. Mol Oral Microbiol. 2024;39(2):29‐39. 10.1111/omi.12432 37718958 PMC10939983

[34] Baima G , Romandini M , Citterio F , Romano F , Aimetti M . Periodontitis and accelerated biological aging: a geroscience approach. J Dent Res. 2022;101(2):125‐132. 10.1177/00220345211037977 34609209

[35] Aquino‐Martinez R . The emerging role of accelerated cellular senescence in periodontitis. J Dent Res. 2023;102(8):854‐862. 10.1177/00220345231154567 36908187

[36] Guo S , Fu L , Yin C , et al. ROS‐induced gingival fibroblast senescence: implications in exacerbating inflammatory responses in periodontal disease. Inflammation. 2024;47(6):1918‐1935. 10.1007/s10753-024-02014-5 38630168

[37] Aquino‐Martinez R , Eckhardt BA , Rowsey JL , et al. Senescent cells exacerbate chronic inflammation and contribute to periodontal disease progression in old mice. J Periodontol. 2021;92(10):1483‐1495. 10.1002/JPER.20-0529 33341947 PMC8281492

[38] Aquino‐Martinez R , Khosla S , Farr JN , Monroe DG . Periodontal disease and senescent cells: new players for an old oral health problem?. Int J Mol Sci. 2020;21(20):7441. 10.3390/ijms21207441 33050175 PMC7587987

[39] Martin OCB , Frisan T . Bacterial genotoxin‐induced DNA damage and modulation of the host immune microenvironment. Toxins. 2020;12(2):63. 10.3390/toxins12020063 31973033 PMC7076804

[40] Iida Y , Honda K , Suzuki T , et al. Brain abscess in which Porphyromonas gingivalis was detected in cerebrospinal fluid. Br J Oral Maxillofac Surg. 2004;42(2):180. 10.1016/S0266-4356(03)00190-6 15013561

[41] Yoo JR , Heo ST , Kim M , Lee CS , Kim YR . *Porphyromonas gingivalis* causing brain abscess in patient with recurrent periodontitis. Anaerobe. 2016;39:165‐167. 10.1016/j.anaerobe.2016.04.009 27085200

[42] Joseph S , Aduse‐Opoku J , Hashim A , et al. A 16S rRNA gene and draft genome database for the murine oral bacterial community. mSystems. 2021;6(1):e01222‐20. 10.1128/mSystems.01222-20 33563782 PMC7883545

[43] Jiang M , Ge Z , Yin S , et al. Cathepsin B modulates Alzheimer's disease pathology through SAPK/JNK signals following administration of *Porphyromonas gingivalis*‐derived outer membrane vesicles. J Clin Periodontol. 2025;52(3):434‐456. 10.1111/jcpe.14109 39726227

