# Supplementary material for: Aging and periodontitis increase brain dissemination of oral bacteria
Source: J Periodontol. 2026 Apr 13;97(9):1965–79. doi: 10.1002/jper.70118 (PMC13568886; doi:10.1002/jper.70118)
Supplement: Supplementary file 1 [file JPER-97-1965-s001.docx]

**Supplemental Table 2.** Pairwise PERMANOVA results

|  |  | **Sample size** | **Permutations** | **pseudo-F** | **p-value** | **q-value** |
| --- | --- | --- | --- | --- | --- | --- |
| **Group 1** | **Group 2** |  |  |  |  |  |
| **Brain Old Perio** | **Brain Young Perio** | 10 | 999 | 4.556.033 | 0.007 | 0.010909 |
|  | **Ligature Old** | 12 | 999 | 7.293.151 | 0.003 | 0.010909 |
|  | **Ligature Young** | 10 | 999 | 4.330.145 | 0.006 | 0.010909 |
|  | **Stool Old Perio** | 12 | 999 | 10.300.234 | 0.004 | 0.010909 |
|  | **Stool Young Perio** | 12 | 999 | 13.128.913 | 0.001 | 0.010909 |
| **Brain Young Perio** | **Ligature Old** | 10 | 999 | 2.808.908 | 0.029 | 0.031071 |
|  | **Ligature Young** | 8 | 999 | 1.849.511 | 0.026 | 0.030000 |
|  | **Stool Old Perio** | 10 | 999 | 4.578.143 | 0.008 | 0.010909 |
|  | **Stool Young Perio** | 10 | 999 | 5.647.455 | 0.007 | 0.010909 |
| **Ligature Old** | **Ligature Young** | 10 | 999 | 1.530.138 | 0.308 | 0.308000 |
|  | **Stool Old Perio** | 12 | 999 | 6.474.040 | 0.003 | 0.010909 |
|  | **Stool Young Perio** | 12 | 999 | 9.503.338 | 0.003 | 0.010909 |
| **Ligature Young** | **Stool Old Perio** | 10 | 999 | 3.577.058 | 0.008 | 0.010909 |
|  | **Stool Young Perio** | 10 | 999 | 5.157.811 | 0.005 | 0.010909 |
| **Stool Old Perio** | **Stool Young Perio** | 12 | 999 | 4.870.746 | 0.023 | 0.028750 |
